# Precision Adjuvant Strategies in Vaccine Development for Substance Use Disorders: Variability and Mechanistic Insights

**DOI:** 10.3390/pharmaceutics17091223

**Published:** 2025-09-20

**Authors:** Yuanzhi Bian, Qiaoqiao Ci, Xin M. Luo, Chenming Zhang

**Affiliations:** 1 Department of Biological Systems Engineering, College of Engineering & College of Agriculture and Life Sciences, Virginia Tech, Blacksburg, VA 24061, USA; yuanzhi8@vt.edu (Y.B.); qiaoc@vt.edu (Q.C.); 2 Department of Biomedical Sciences and Pathobiology, Virginia-Maryland College of Veterinary Medicine, Virginia Tech, Blacksburg, VA 24061, USA; xinluo@vt.edu

**Keywords:** vaccine adjuvant, substance use disorder, aluminum salt, emulsion adjuvant, toll-like receptor agonist, protein-based adjuvant, cytokine modulator, precision vaccinology, nanoparticle vaccine, opioid vaccine

## Abstract

Substance use disorders (SUDs) remain a major global health challenge with limited treatment options and high relapse rates. Vaccines that induce drug-sequestering antibodies have shown promise, but their efficacy is hindered by the poor immunogenicity of small-molecule haptens. Adjuvants, substances that enhance immune responses, are critical for overcoming this limitation and improving vaccine efficacy. This review synthesizes over two decades of preclinical and clinical research to guide rational adjuvant design for SUD vaccines. Five major adjuvant classes are examined: aluminum-salt adjuvants, emulsion adjuvants, toll-like receptor (TLR) agonists, protein immunopotentiators, and cytokine modulators. Their physicochemical properties, innate immune activation profiles, and applications in nicotine, stimulant, and opioid vaccines are discussed. Comparative analyses reveal pronounced drug-specific and carrier-specific variability. Case studies illustrate the superior performance of a complementary TLR-agonist pair in a nicotine nanovaccine versus its limited effect in oxycodone vaccines. They also reveal the differential efficacy of an oil-in-water emulsion adjuvant across antigen types. Four principles emerge: (i) no adjuvant is universally optimal; (ii) drug pharmacology influences immune signaling; (iii) adjuvant-carrier compatibility is important; (iv) complementary adjuvant pairings often outperform single agents. These insights support a precision-vaccinology paradigm that tailors adjuvant strategies to each drug class and the delivery vehicle, advancing the development of next-generation SUD vaccines.

## 1. Introduction

Substance use disorder (SUD) is a chronic mental health condition characterized by compulsive drug seeking and continued use despite harmful consequences [1]. Substances associated with the disorder include but are not limited to alcohol, marijuana, tobacco, cocaine, methamphetamine, and opioid compounds [2]. Collectively, SUDs arising from this broad spectrum of drugs constitute a substantial global public-health burden, driving significant morbidity, mortality, and disability [3,4,5]. According to the 2023 United Nations Office on Drugs and Crime (UNODC) World Drug Report, an estimated 39.5 million people worldwide meet the diagnostic criteria for an SUD [6]. At least 585,000 deaths occur globally each year as a direct consequence of drug consumption, and roughly two-thirds of these fatalities involve opioids [7]. In the United States, provisional data from the Centers for Disease Control and Prevention (CDC) reveal approximately 114,000 drug overdose fatalities in the 12-month period ending June 2023, the highest per capita overdose death rate ever recorded [8]. Beyond fatalities, the societal costs of SUDs encompass lost productivity, diminished quality of life, family disruption, and criminal justice expenditures [9]. Adapting the method used by researchers at the CDC, the Joint Economic Committee estimated that opioid use disorder (OUD) and overdose alone costed the United States nearly USD1.5 trillion in 2020, representing a 37% increase from 2017 [10].

Despite decades of research, treatment options remain limited. For OUD, medications that include methadone, buprenorphine, and extended-release naltrexone represent the cornerstone of evidence-based care [11]. When taken as prescribed, methadone and buprenorphine reduce all-cause mortality by up to 50% [12]. However, their real-world effectiveness is curtailed by daily supervised dosing requirements, regulatory barriers that restrict prescriber capacity, diversion risk, and the persistence of stigma among patients and providers [13,14,15]. Extended-release naltrexone circumvents some of these limitations by antagonizing the μ opioid receptor without triggering rewarding effects [16]. Nonetheless, its initiation requires a period of opioid abstinence that many individuals find intolerable [17]. In addition to OUD, stimulant use disorders involve centrally acting drugs such as cocaine, methamphetamine, and prescription amphetamines, which stimulate the nervous system and increase alertness, attention, and energy [18,19]. Cannabis use disorders refer to problematic patterns of marijuana or related product use. Cannabis acts primarily as a psychoactive drug producing euphoria, altered perception, and relaxation [20]. For both stimulant and cannabis use disorders, pharmacological therapies remain experimental and produce only modest reductions in craving or relapse [21,22,23,24,25,26]. Behavioral interventions such as contingency management, cognitive behavioral therapy, and motivational enhancement therapy offer additional benefit, but demand sustained engagement and intensive resources [27,28,29,30,31,32]. Across drug classes, meta-analyses reveal relapse rates that routinely exceed 60% within the first year of treatment discontinuation [33,34,35,36].

In light of these limitations, active immunotherapy has emerged as a compelling alternative. Unlike small-molecule antagonists that compete for receptor occupancy [37], vaccines operate through a pharmacokinetic mechanism. They induce high-affinity antibodies that bind the target drug in the periphery [38]. These antibody–drug complexes are too large to cross the blood–brain barrier, thereby blunting psychoactive effects. The vaccine approach offers several advantages, including the absence of intrinsic abuse liability and longer duration of therapeutic action [39,40]. Additionally, because antibodies act upstream of central receptors, vaccines can theoretically be combined with existing medications without pharmacodynamic conflict [41]. Early proof of concept appeared in the 1970s when Bonese and colleagues showed that rhesus monkeys immunized with a morphine–protein conjugate self-administered less heroin [42]. The modern era of vaccines targeting SUDs is marked by the integration of rational hapten design [43,44,45], potent carrier proteins [46,47], and a renewed emphasis on adjuvant innovation [48,49]. These advances in formulation have enabled multiple vaccine candidates to progress to clinical trials [50,51,52], reflecting steady improvement in immunogenicity and translational potential.

**Figure 1:**
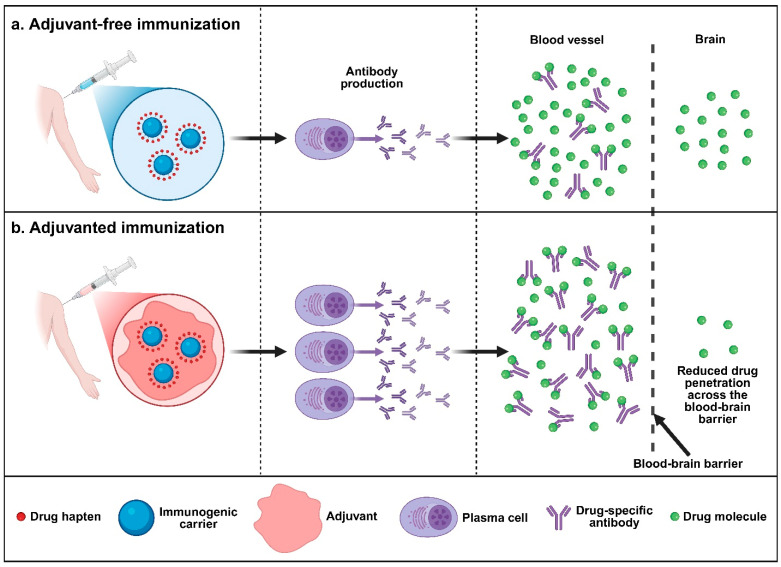
Adjuvants enhance the immunogenicity and protective efficacy of SUD vaccines. (**a**). Adjuvant-free immunization elicits a modest drug-specific antibody response and affords limited protection. (**b**). By contrast, adjuvanted immunization induces robust antibody production and more effectively prevents drug entry into the brain. This figure was created in BioRender.com.

Among all formulation variables (hapten structure, carrier protein, dosing schedule), the choice of adjuvant has proven highly influential [53,54]. Adjuvants are immunostimulatory substances co-administered with antigens to enhance vaccine-induced immunity [55,56,57]. By increasing antigen uptake and presentation, activating innate signaling pathways, and shaping cytokine release, adjuvants are indispensable for eliciting robust immune responses, especially for the weakly immunogenic, small-molecule haptens of psychoactive substances. Enhanced immunogenicity elicited by adjuvanted vaccines translates into superior protection against the target drug (Figure 1). Aluminum hydroxide-based adjuvant alone sufficed to advance NicVAX® into Phase III clinical trials, but the vaccine fell short of primary efficacy endpoints because only a minority of subjects attained or maintained abstinence for a prespecified period during or after the study [58,59,60]. Subsequent preclinical studies demonstrated that pairing aluminum salts with toll-like receptor (TLR) agonists significantly enhanced the immunogenicity and efficacy of opioid vaccines [61,62]. Another preclinical study revealed that vaccines targeting fentanyl produced robust anti-fentanyl antibody titers when formulated with protein adjuvants derived from heat-labile enterotoxins from *E. coli* (LT) [63]. These included the A1 domain of LT (LTA1) and the double-mutant LT, known as dmLT or LT(R192G/L211A). In contrast, aluminum salts primarily promoted antibody responses against the carrier protein rather than fentanyl. These findings suggest that adjuvant efficacy is likely drug-specific and can be shaped by a combination of factors, such as adjuvant physicochemical and immunological properties, innate receptor engagement, and drug-induced modulation of immune pathways.

A comprehensive understanding of these variables is now critical for advancing next-generation SUD vaccines from bench to clinic. Therefore, this review has three aims. First, it catalogs the principal adjuvant categories, including aluminum salt-based adjuvants, emulsion adjuvants, TLR agonists, protein adjuvants, and cytokine modulators, employed across SUD vaccine candidates. Second, it summarizes the efficacy of these adjuvants across different drug targets, highlighting through comparative data how their efficacy can vary substantially depending on the substance. Third, it explores the underlying mechanisms that may account for this variability, highlighting the roles of drug-receptor interactions, immune signaling pathways, and adjuvant physicochemical properties. Collectively, this precision-adjuvant perspective provides insights for rational adjuvant selection, advocating that tailored adjuvant strategies are indispensable for the development of effective vaccines against SUDs. 

To clarify scope, this review provides in-depth analysis for vaccines against nicotine, cocaine, methamphetamine, and opioids, where adjuvant-focused vaccine studies enable meaningful cross-study comparisons. In contrast, alcohol and cannabis currently lack a sufficiently developed adjuvant literature to support the same level of analysis. For alcohol, the physicochemical properties of ethanol (e.g., very small, poorly immunogenic) have limited progress with vaccine development [64], resulting in few adjuvant-centric datasets. For cannabis, adjuvant-defined studies remain limited, with only two preclinical vaccine reports [65,66], both employing the same adjuvants (aluminum salt-based adjuvant + the TLR agonist CpG oligodeoxynucleotide), which precludes a comparative assessment of adjuvant choice. Accordingly, we briefly note these efforts but reserve in-depth analysis for drug classes with a stronger evidence base. Unless otherwise indicated, conclusions about relative adjuvant performance are derived primarily from preclinical rodent studies. Human data remain limited and mixed, as illustrated by the NicVAX® and TA-CD cocaine vaccine trials.

## 2. Adjuvant Platforms in SUD Vaccinology

This section provides an overview of the major adjuvant platforms that have been explored in the development of vaccines targeting SUDs. These platforms include traditional aluminum salt-based adjuvants and emulsion-based adjuvant systems, as well as strategies such as toll-like receptor agonists, protein-based adjuvants, and cytokine modulators. For each adjuvant class, we summarize chemical composition, mechanisms of action, and the immunological profiles observed in both preclinical and clinical studies. By highlighting the breadth of adjuvant technologies and their unique immunostimulatory properties, this section sets the stage for understanding how adjuvant selection can profoundly influence the immune efficacy and translational success of SUD vaccines.

### 2.1. Aluminum Salt-Based Adjuvants

Aluminum-salt (Alum) adjuvants, including formulations based on aluminum hydroxide and aluminum phosphate, are the most widely used vaccine adjuvants to date [67,68,69]. The material commonly referred to as aluminum hydroxide (AH) adjuvant is, in fact, aluminum oxyhydroxide (AlOOH), an amorphous precipitate formed by adding sodium hydroxide to aluminum salts under controlled conditions [68,70]. This AlOOH matrix avidly adsorbs antigen, persists at the injection site as a depot, and induces a T helper 2 (Th2) cell-associated immune response by facilitating antigen internalization by antigen-presenting cells (APCs) [71]. In addition, AH adjuvant promotes the release of damage-associated molecular patterns (DAMPs) such as uric acid that activates pattern recognition receptors (PRRs). Aluminum phosphate (AP) adjuvant, on the other hand, is composed of aluminum hydroxyphosphate, Al(OH)_×_(PO_4_)_y_, in which a proportion of the hydroxyl groups of aluminum hydroxide are replaced by phosphate groups [68,72]. Despite being chemically different, the action mechanism of AP adjuvant is similar to that of AH adjuvant. AP adjuvant adsorbs antigen and accumulates at the injection site upon administration, and this depot effect allows the antigen to be slowly released, facilitating antigen uptake by APCs [73]. Studies have also shown that both AH and AP adjuvants activate the nucleotide-binding oligomerization domain (NOD)-like receptor protein 3 (NLRP3) inflammasome in APCs [74,75,76]. Activation of the NLRP3 inflammasome leads to the secretion of pro-inflammatory cytokines such as IL-1β, which stimulates the maturation and differentiation of CD4+ T cells.

Alum’s long clinical history and manufacturing simplicity make it the default starting point for the development of new vaccines. A summary of SUD vaccines employing Alum adjuvants is provided in Table 1. Alum adjuvants have been used extensively in nicotine and cocaine vaccines, some of which advanced to Phase III clinical trials as mentioned previously. Alum have also been explored in vaccines against methamphetamine (METH) and OUDs. For instance, Rüedi-Bettschen et al. showed that an AH-adjuvanted conjugate vaccine (IC_KLH_-SMO9) effectively reduced METH-induced impairment of behavioral responding for food in rats [77]. Kosten et al. tested a morphine conjugate vaccine (KLH-6-SM) adjuvanted with AH gel in rats and showed that the vaccine elicited a prominent anti-morphine antibody response [78]. The vaccine also reduced morphine distribution to the brain and attenuated its behavioral effects. Pravetoni et al. demonstrated that a conjugate vaccine consisting of an oxycodone hapten and the carrier protein keyhole limpet hemocyanin (KLH) (6OXY(Gly)_4_-KLH) increased drug binding in circulation, reduced brain penetration, and blunted analgesia for both oxycodone and hydrocodone [79]. In a subsequent study, Walter et al. showed that formulating the oxycodone conjugate vaccine onto a lipid-poly(lactic-*co*-glycolic) acid (PLGA) hybrid nanoparticle further enhanced its immunogenicity and pharmacokinetic performance [80]. When paired with AH adjuvant, the nanoparticle-based vaccine elicited higher antibody titers and superior drug-neutralizing capacity than formulations adjuvanted with the TLR ligands resiquimod (R848) and monophosphoryl lipid A (MPLA). Alum adjuvants have also been incorporated into multivalent constructs against SUDs. de Villiers et al. established a trivalent nicotine vaccine formulated with three distinct nicotine immunogens and adjuvanted with Alum [81]. The trivalent nicotine vaccine demonstrated superior immunogenicity and pharmacokinetic efficacy compared to monovalent vaccines. Song and colleagues formulated multivalent vaccines that simultaneously target multiple substances, such as fentanyl, carfentanil, oxycodone, heroin, METH, and their analogs or metabolites [82]. They found that the multivalent formulation induced independent antibody responses against the respective targets in both mice and rats, effectively blocking the penetration of individual drugs into the brain. 

While Alum adjuvants remain an appealing choice for initial vaccine development because of their regulatory acceptance and cost, their immunological profile is limited and may not be sufficient to confer broad or robust protection against all drug targets [85,86]. In addition to Alum, emulsion-based adjuvants have attracted substantial interest due to their proven ability to promote strong and durable immune activation [87]. In the following subsection, we will explore the composition, mechanisms, and application of emulsion adjuvants in SUD vaccine development.

### 2.2. Emulsion-Based Adjuvants

Emulsion-based adjuvants are typically oil-in-water (O/W) or water-in-oil (W/O) formulations that enhance immune responses by creating antigen depots and stimulating innate immune activation [56,88]. These formulations include but are not limited to Freund’s adjuvant, Sigma Adjuvant System®, MF59, and AS03. Freund and colleagues invented the first W/O emulsion adjuvant in the 1940s [89]. It was composed of killed tubercle bacilli suspended in paraffin oil, combined with horse serum and a lanolin-like substance (Aquaphor). However, Freund’s adjuvant is not approved for use in human vaccines due to safety concerns related to its toxicity and local reactogenicity. An alternative to Freund’s adjuvant is Sigma Adjuvant System® (SAS), which is a stable O/W emulsion derived from bacterial and mycobacterial cell walls [90]. MF59 and AS03 are squalene-based O/W emulsions approved for use in licensed human vaccines [69,91,92]. They are known to rapidly recruit immune cells, elevate cytokine secretion, facilitate antigen uptake and trafficking to lymph nodes, and promote T cell priming. It was also found that MF59 triggers rapid adenosine triphosphate (ATP) release at the injection site [93]. This extracellular ATP is a danger signal linking local tissue responses to enhanced adaptive immunity. Mechanistically, emulsion adjuvants facilitate inflammasome activation, enhance antigen retention, promote T follicular helper (Tfh) cell responses, and support germinal center formation, which are all crucial for strong humoral immunity [94,95].

A growing body of evidence supports the use of emulsion-based adjuvants in vaccines targeting SUDs. Key emulsion-adjuvanted formulations and their performance across SUD targets are outlined in Table 2. Freund’s adjuvant has been widely used in preclinical research on vaccines targeting SUDs since as early as in the 1970s. Torten et al. demonstrated that immunization with a fentanyl-bovine gamma globulin (BGG) conjugate vaccine adjuvanted with Freund’s adjuvant effectively neutralized the analgesic effect of fentanyl in mice and dogs [96]. Mice administered with the fentanyl vaccine exhibited significantly reduced responses in the hot plate assay. Passive transfer of anti-fentanyl serum similarly prevented fentanyl-induced analgesia. In dogs, anti-fentanyl antibodies blocked fentanyl-induced alterations in cardiovascular and respiratory parameters, highlighting the potential of immunopharmacological strategies for mitigating opioid toxicity. Later on in the 1990s, Fox et al. evaluated a cocaine vaccine composed of a cocaine-bovine serum albumin (BSA) conjugate adjuvanted with Freund’s adjuvant in mice [97]. Immunization with the cocaine vaccine elicited high-titer, long-lasting antibody responses that significantly reduced brain cocaine levels. Passive immunization with an anti-cocaine monoclonal antibody similarly blocked cocaine self-administration in rats. These findings suggest that both active and passive immunization strategies effectively altered cocaine pharmacokinetics and attenuated drug reinforcement behaviors in rodent models. In the 2010s, Pravetoni and colleagues evaluated a nicotine vaccine using 3′-aminomethylnicotine conjugated to recombinant *Pseudomonas* exoprotein A (3′-AmNic-rEPA), mixed with Freund’s adjuvant, in a rat model [98]. The vaccine significantly reduced brain nicotine concentrations by 90% following brief smoke exposure (10 min nose-only) and by 35% after prolonged exposure (2 h whole-body). These results demonstrated robust antibody-mediated nicotine sequestration. It was also shown that passive immunization with the monoclonal antibody Nic311 resulted in comparable effects, indicating the importance of systemic antibody responses in mitigating nicotine brain distribution following cigarette smoke inhalation. 

In addition to Freund’s adjuvant, the other emulsion adjuvants have also been explored in vaccines against SUDs. Moreno et al. evaluated six structurally distinct METH haptens using SAS to develop an effective vaccine [99]. Three hapten candidates, MH2(R), MH6, and MH7, elicited high antibody titers (45–220 μg/mL) and nanomolar affinity for (+)-METH (82–169 nM). MH2(R) included a conformational constraint designed to enhance mimicry of METH’s most stable structure. Miller et al. investigated the efficacy of METH vaccines formulated with SAS to attenuate drug-induced physiological and behavioral effects in rats [100]. Among the three vaccine candidates tested (MH2(R), MH6, and MH7), MH6 induced the highest antibody titers with strong nanomolar affinity for METH. It effectively sequestered the drug in serum and reduced its distribution to the brain. MH6 vaccination also significantly mitigated METH-induced hyperthermia, hypothermia, and locomotor disruptions, demonstrating vaccine-induced functional protection. Collins and Janda investigated hapten clustering as a strategy to enhance nicotine vaccine efficacy [101]. Using SAS as the adjuvant, they evaluated monovalent and trivalent nicotine haptens conjugated to ovalbumin. The trivalent hapten with a diglycine linker, triAM1(Gly)_2_, elicited antibody responses comparable to the monovalent AM1 (a 3′-substituted nicotine hapten), despite having a lower hapten density. Additionally, the monovalent AM1(Gly)_2_ produced even higher antibody affinity and concentration than AM1. These findings demonstrated that both hapten clustering and linker optimization can enhance vaccine efficacy. Another study by Robinson et al. evaluated the efficacy of an oxycodone conjugate vaccine formulated with either AH adjuvant or MF59 in mouse models [103]. The key findings showed that AH-adjuvanted vaccines elicited higher oxycodone-specific IgG titers and greater early expansion of germinal center B cells and Tfh cells. They were more effective than vaccines incorporating MF59 in blocking oxycodone-induced motor activity and brain penetration in adult mice. However, in aged mice, AH and MF59 performed comparably, suggesting that age influences adjuvant responsiveness. Moreno et al. investigated nicotine vaccines composed of AM1 conjugated to various carrier proteins and adjuvanted with AS03 [104]. The vaccines led to strong anti-nicotine antibody responses in mice and rats, with the AM1-tetanus toxoid (TT) formulation outperforming the other carrier proteins explored, namely KLH and cross-reactive material 197 (CRM_197_). In vaccinated rats undergoing intravenous nicotine self-administration, increased nicotine intake and motivation to self-administer were observed. These results demonstrated compensatory behavior because of reduced nicotine brain penetration, supporting the vaccine’s efficacy despite high drug exposure.

Emulsion-based adjuvants offer versatility and have established safety profiles in licensed vaccines [105]. Their demonstrated efficacy in preclinical SUD models further supports their potential for translational development. In the next subsection, we will examine toll-like receptor agonists, another mechanistically distinct and widely used class of immunostimulatory adjuvants, which can be used alone or in combination with emulsions to enhance vaccine efficacy.

### 2.3. Toll-like Receptor Agonists

Toll-like receptors (TLRs) are a family of pattern recognition receptors (PRRs) that serve as the primary sensors of innate immunity. They recognize distinct structural motifs related to pathogens or components of host cells released during cell damage, often referred to as pathogen-associated molecular patterns (PAMPs) or damage-associated molecular patterns (DAMPs), respectively [106,107]. TLRs are expressed on immune cells (including dendritic cells, macrophages, granulocytes, T cells, B cells, natural killer cells, and mast cells), endothelial and epithelial cells, as well as tumor cells. Some TLRs are present on the plasma membrane (TLR1, 2, 4, 5, and 6), while the others are located within the endoplasmic reticulum (TLR3, 7, 8, and 9) and rapidly recruited to endosomal–lysosomal compartments upon pathogen invasion or host cell death [108,109]. Upon TLR agonist binding, either the myeloid differentiation factor 88 (MyD88)-dependent or the toll/IL-1R domain-containing adaptor-inducing IFN-β (TRIF)-dependent signaling pathway is activated [110,111,112]. The pathways lead to the activation of transcription factors, including nuclear factor-kappa B (NF-κB) or interferon-regulatory factors (IRFs), to modulate the expression of cytokines and chemokines, such as type I interferons. Because of their immunomodulatory activities, TLR agonists have been used extensively in vaccines against SUDs. 

The application of TLR agonists across nicotine, opioid, and stimulant vaccine platforms is detailed in Table 3. In a nicotine vaccine study using a mouse model, Zhao et al. engineered a lipid-PLGA hybrid nanoparticle platform, which allows for the co-delivery of nicotine immunogen and molecular adjuvants [49]. The TLR agonists MPLA (TLR4 agonist), R848 (TLR7/8 agonist), CpG oligodeoxynucleotide 1826 (CpG ODN 1826, a TLR9 agonist), and their combinations were encapsulated into the nanoparticle. Immunological and pharmacokinetic evaluation revealed that the nanovaccine incorporating MPLA and R848 showed the best efficacy. Matyas et al. inserted MPLA into liposomes (L(MPLA)), which were used as the adjuvant for heroin conjugate vaccines [113]. They tested two formulation strategies: conjugating haptens to a peptide carrier embedded in the outer surface of L(MPLA) or to TT mixed with the liposomes. It was found that both approaches elicited anti-heroin antibodies in mice, with the TT-based formulation producing a higher antibody titer. TLR agonists have been used alongside Alum to increase the desired immune response, as they stimulate different immune pathways and may provide additive or synergistic effects depending on the context. Bremer et al. developed a fentanyl vaccine (Fent-TT), formulated with AH adjuvant and CpG ODN 1826 [114]. The vaccine elicited high-affinity antibodies capable of neutralizing fentanyl and its analogs in mice. Fent-TT significantly shifted opioid dose–response curves, reduced brain fentanyl levels, and provided strong protection against overdose. Bremer and colleagues also constructed a clinically viable heroin conjugate vaccine using a rationally optimized heroin-TT immunoconjugate formulated with Alum and CpG ODN adjuvants [115]. The vaccine induced strong and durable anti-heroin antibody responses in both mice and rhesus monkeys. It significantly reduced heroin potency (>15-fold in mice and >4-fold in monkeys) and generated antibodies with nanomolar affinity for 6-acetylmorphine, the primary psychoactive heroin metabolite. These preclinical findings support the vaccine’s potential in treating OUD by sequestering heroin and its metabolites in the periphery and preventing central effects. Kimishima et al. investigated how different carrier-adjuvant combinations influence the efficacy of an anti-cocaine vaccine using a pharmacokinetic approach [116]. The authors demonstrated that combining the TLR9 agonist CpG ODN 1826 with Alum significantly enhanced antibody titers, cocaine sequestration, and behavioral blockade, outperforming TLR5 activation alone by flagellin (FliC). While FliC offered dual TLR5 activation and carrier potential, its efficacy was limited compared to TT. Crouse et al. demonstrated that the incorporation of the TLR7/8 agonist INI-4001 with Alum significantly increased fentanyl-specific antibody titers, enhanced protection against respiratory depression and overdose, and attenuated fentanyl self-administration in rodent and porcine models [62]. Hossain et al. reported the development and evaluation of two METH vaccine candidates that use monoamine or diamine peptide linkers conjugated to oxidized mannan (OM), a poly-mannose immunogenic carrier [117]. In a mouse model, both OM-conjugated vaccines produced METH-specific antibody responses. The diamine linker-based vaccine (Vaccine 2) was further enhanced by the inclusion of CpG ODNs and AH adjuvant. Haile et al. reported a METH vaccine consisting of a succinyl-METH hapten conjugated to TT and adjuvanted with AH adjuvant and the TLR5 ligand entolimod [118]. The vaccine elicited high anti-METH antibody titers in mice and significantly attenuated METH-induced locomotor activity. Passive immunization with anti-METH antibodies suppressed METH-induced increases in mean arterial pressure in rats and prevented reinstatement of drug-seeking behavior. Barrientos et al. developed a bivalent conjugate vaccine targeting heroin and fentanyl [119]. The vaccine was composed of two distinct hapten-protein conjugates (TT-6-AmHap and TT-*para*-AmFenHap) and adjuvanted with Army Liposome Formulation containing MPLA and AH adjuvant. It induced high IgG titers with nanomolar affinity for heroin, 6-acetylmorphine, morphine, and fentanyl, and effectively blocked the antinociceptive effects of each drug in mice, including a heroin/fentanyl mixture. Importantly, the vaccine showed no interference with therapeutic opioids (methadone, buprenorphine) or naloxone, supporting its potential as a complementary strategy for opioid overdose prevention. Lockner et al. evaluated liposomal formulations of a nicotine vaccine (AM1-KLH) in mice [120]. They revealed that incorporating the TLR4 agonist MPLA, either alone (LP4) or with a TLR2 agonist Pam3CAG (LP24), significantly enhanced anti-nicotine antibody titers and affinities compared to traditional adjuvants like SAS. Liposomes without TLR agonists (LP0) or with the TLR2 agonist only (LP2) were much less effective, highlighting the crucial role of TLR4 signaling in boosting vaccine immunogenicity. These results support the application of liposomal adjuvants, particularly MPLA-based formulations, to improve the efficacy of nicotine vaccines. Powers et al. reported the development of a self-adjuvanting fentanyl vaccine by co-conjugating the TLR7/8 agonist UM-3006 and a fentanyl hapten onto the carrier protein CRM_197_ [121]. Compared to admixtures of unconjugated components, this co-conjugation strategy significantly enhanced fentanyl-specific IgG titers, promoted a Th1-biased immune response, and provided superior protection against fentanyl-induced analgesia and respiratory depression in mice. Addition of Alum further improved antibody titers and serum fentanyl sequestration. Hwang et al. explored heroin vaccine formulations using TLR9 (CpG ODN 1826) and TLR3 (double-stranded RNA) agonists, both with Alum [122]. It was found that the adjuvants enhanced anti-heroin antibody responses and attenuated heroin-induced antinociception in mice. Notably, the CpG and Alum formulation but not the TLR3 formulation conferred protection from lethal heroin doses and maintained potency when stored as a lyophilized solid or as a liquid for over 30 days. In a study conducted by Stevens et al., a METH vaccine (IC_KLH_-SMO9) formulated with the TLR4 agonist GLA-SE, a synthetic lipid A in the form of an O/W emulsion, produced significantly higher antibody titers and affinities in mice compared to traditional Alum adjuvants [123]. 

In addition to TLR agonists, protein-based adjuvants have garnered increasing attention in recent years as distinctive immunostimulatory agents. While protein-based adjuvants often interact with TLRs (e.g., flagellin is a TLR5 agonist) [124], they also engage in different mechanisms of immune activation. These adjuvants, many of which are derived from bacterial enterotoxins or engineered fusion constructs, offer the potential to augment immune responses to extend the protective effects of SUD vaccines. In the next subsection, we examine the immunological features, examples, and emerging applications of protein-based adjuvants in the context of SUD vaccine development.

### 2.4. Protein-Based Adjuvants

Protein-based adjuvants represent a versatile and mechanistically rich class of immunostimulatory agents. They are often derived from microbial proteins, bacterial toxins, or synthetic protein constructs engineered to engage the immune system [125]. Unlike small-molecule adjuvants that typically function via defined receptor interactions, protein adjuvants can activate the immune system through multiple pathways, including receptor binding, inflammasome activation, antigen deposition, and enhanced trafficking to lymphoid tissues [125,126,127,128,129]. Moreover, many protein-based adjuvants exhibit physical characteristics, such as multimeric or particulate assembly, that mimic pathogen-like structures, thereby enhancing antigen uptake by APCs and promoting robust germinal center reactions [130]. Importantly, protein-based adjuvants may also serve dual functions as both immunopotentiators and structural carriers, allowing for rational fusion or co-display of antigens, haptens, or T-helper epitopes within the same molecular scaffold [131,132,133]. This co-delivery strategy has been shown to stabilize antigen conformation, increase co-localization of adjuvant and antigen, and improve lymph node targeting. Furthermore, the use of protein-based platforms offers flexibility in tailoring the immune response. They can be engineered to bias toward humoral or cellular immunity or enhance mucosal IgA production depending on their design and route of administration [134,135]. Due to their recombinant nature, protein-based adjuvants are suitable for scalable production under good manufacturing practice (GMP) conditions, with favorable safety profiles for both systemic and mucosal vaccine delivery.

Several protein-based adjuvants have been investigated in vaccines targeting SUDs. Representative examples of protein-based adjuvants used in SUD vaccine research are listed in Table 4. Flagellin, a TLR5 ligand derived from bacterial flagella, has been employed as both the adjuvant and carrier. Lockner et al. investigated the use of recombinant flagellin (FliC) as both a carrier protein and adjuvant for a cocaine vaccine [136]. They chemically conjugated a cocaine hapten (GNE) to FliC and demonstrated that the resulting conjugate vaccine elicited dose-dependent anti-cocaine antibody responses in mice. Notably, when formulated with Alum, GNE-FliC outperformed a benchmark KLH-based vaccine, indicating that flagellin–Alum synergy can further enhance the immunogenicity of the cocaine vaccine. Protein adjuvants derived from heat-labile enterotoxins of *E. coli* have also been explored for systemic and mucosal immunization. Stone et al. evaluated a fentanyl conjugate vaccine in mice, using *E. coli* heat-labile enterotoxin-derived adjuvants (dmLT or LTA1) delivered intramuscularly or via mucosal routes [63]. The researchers found that both dmLT and LTA1 significantly enhanced anti-fentanyl IgG and IgA production and improved protection against fentanyl-induced analgesia. Notably, mucosal booster immunizations (sublingual dmLT or intranasal LTA1) induced strong IgA responses, which correlated with protection, highlighting a previously unrecognized role for IgA in opioid vaccine efficacy. The B subunit of cholera toxin (CTB), another bacterial enterotoxin-derived adjuvant, was utilized in the TA-CD cocaine vaccine [84]. The TA-CD cocaine vaccine was composed of succinylnorcocaine conjugated to CTB and adsorbed onto AH adjuvant. Its safety and efficacy were evaluated in a Phase III randomized, double-blind, placebo-controlled trial on 300 cocaine-dependent individuals. Although the vaccine was safe and two-thirds of the recipients achieved the target antibody level (greater than or equal to 42 μg/mL), there were no statistically significant reductions in cocaine-positive urines across groups. Subjects with high IgG titers demonstrated improved treatment retention and a non-significant trend toward increased late abstinence, suggesting partial efficacy and highlighting the need for strategies that sustain antibody levels and support behavioral change. Sanderson et al. developed a peptide-based nicotine vaccine, in which a nicotine hapten was conjugated to a synthetic peptide containing both a B cell epitope and a conformationally biased C5a receptor agonist as a molecular adjuvant [137]. Rats immunized with the peptide-based vaccine produced nicotine-specific antibodies and exhibited significantly reduced behavioral responses to nicotine in a Pavlovian conditioning task. This study revealed that targeted delivery of antigen and immunostimulatory signals via C5a receptor (C5aR)-bearing APCs can induce protective immunity against nicotine. Furthermore, the use of recombinant peptide carriers with self-adjuvanting properties has shown promise in enhancing vaccine efficacy against SUDs. Rudra et al. reported a fully synthetic, self-adjuvanting cocaine vaccine based on peptide nanofibers [138]. A novel cocaine hapten was conjugated to a self-assembling peptide (KFE8), forming β-sheet-rich nanofibers that elicited robust anti-cocaine antibody responses in mice. Vaccinated mice showed a significant reduction in cocaine-induced hyperactivity, providing evidence that the peptide nanofibers can provide a promising platform for immunotherapy against cocaine use disorder.

Although protein-based adjuvants are less frequently employed, their modularity, safety profiles, and dual functionality as both carrier and adjuvant make them attractive candidates for future translational research. Their ability to engage innate immunity while minimizing inflammatory toxicity aligns with the growing demand for well-tolerated vaccine platforms in vulnerable populations [125]. Nonetheless, in cases where precise immune modulation is needed, especially to offset the immunosuppressive effects of certain drugs or to mediate immune cell polarization state, cytokine modulators have emerged as a complementary or alternative strategy [139,140]. In the following subsection, we will discuss the rationale and application of cytokine-based adjuvants in SUD vaccine development. The focus will be on how they can reshape immune dynamics beyond the capabilities of conventional PRR agonists.

### 2.5. Cytokine Modulators

Cytokines are small, secreted proteins that play a central role in cell-to-cell communication and the regulation of immune responses [141]. They encompass multiple functional classes, including but not limited to interleukins, interferons, and tumor necrosis factors, each contributing to distinct aspects of immune activation, inflammation, and homeostasis [142,143]. Interleukins (ILs) are a large and diverse group of cytokines primarily responsible for mediating communication between leukocytes [144,145,146,147,148,149]. They regulate a variety of processes, such as T cell differentiation, B cell proliferation, and the activation of macrophages and dendritic cells. Interferons (IFNs) are primarily involved in antiviral defense and immunoregulation [150]. Type I IFNs (e.g., IFN-α, IFN-β) are rapidly secreted by infected cells and act through the Janus kinase (JAK)-signal transducer and activator of transcription (STAT) signaling pathway to induce the expression of interferon-stimulated genes (ISGs), which inhibit viral replication and activate APCs [151]. IFN-γ, the only type II interferon, is primarily produced by activated T cells and NK cells in response to certain antigens or other cytokines [152]. It plays an important role in immune regulation, especially in the activation and differentiation of immune cells. In addition, IFN-γ upregulates the expression of major histocompatibility complex (MHC) molecules in dendritic cells, a major class of APCs, therefore promoting antigen presentation to T cells [153,154]. Flow cytometry analyses suggest that IFN-γ could exert additive or synergistic effects with TLR agonists in promoting dendritic cell activation, underscoring its promise as an immunoadjuvant [155]. Tumor necrosis factors (TNFs) are important inflammation mediators. For instance, secreted predominantly by macrophages, TNF-α binds to TNF receptors (TNFR1 and TNFR2) and activates signaling cascades such as NF-κB and the mitogen-activated protein kinase (MAPK) signaling pathways, stimulating the production of pro-inflammatory cytokines [156,157,158]. Collectively, cytokines function as a finely tuned network that orchestrates immune responses through both stimulatory and regulatory pathways. These immunomodulatory activities make them potent tools for immunotherapy and vaccine adjuvant design.

Although many cytokines have been approved as therapeutics and are being actively investigated as adjuvants in immunotherapies for cancer and infectious diseases [159,160,161], their application in vaccines targeting SUDs remains rare. Emerging uses of cytokine-based immune modulation in SUD vaccine design are summarized in Table 5. Preliminary observations from our group indicate that co-delivery of IFN-γ with the TLR agonist R848 or polyinosinic:polycytidylic acid (poly I:C, a TLR3 agonist) in a nanoparticle-based oxycodone vaccine markedly enhances the generation of high-affinity anti-oxycodone antibodies in mice, compared to Alum-based formulations. (These findings are preliminary and should be interpreted with caution until independently replicated.). The antibodies effectively sequestered oxycodone in circulation and limited its distribution to the brain following drug challenge. As a result, the vaccine markedly attenuated oxycodone-induced antinociception, as demonstrated by the hot plate assay. Interestingly, although cytokines have not been widely applied as adjuvants in SUD vaccine development, emerging studies suggest that blocking suppressive cytokines can be equally powerful in enhancing vaccine efficacy. Blocking suppressive cytokines relieves immunoregulatory constraints and promotes more robust immune responses. Laudenbach et al. demonstrated that blocking IL-4 signaling with a neutralizing monoclonal antibody significantly enhanced the efficacy of a conjugate vaccine against oxycodone (OXY-KLH) [162]. Mice vaccinated with OXY-KLH plus anti-IL-4 monoclonal antibody produced higher levels of IgG2a and IgG3, showed reduced brain penetration of oxycodone, and exhibited diminished toxic and behavioral effects, including respiratory depression and analgesia. This approach was effective across multiple immunization routes, showed no major safety concerns, and has been generalized to other vaccine platforms. These findings suggest that IL-4 modulation is a promising strategy for improving vaccines against opioids and other indications. Similarly, Crouse et al. demonstrated that neutralizing IL-4 significantly enhanced the efficacy of vaccines targeting opioids such as oxycodone and fentanyl in mice [163]. Administration of an anti-IL-4 monoclonal antibody alongside the opioid vaccines yielded increased anti-opioid antibody titers, improved germinal center formation in secondary lymphoid organs, and reduced opioid distribution to the brain. These effects were not replicated by genetic deletion of IL-4 receptors or STAT6, suggesting that active IL-4 signaling, rather than its downstream components, is critical. Crouse et al. also investigated the role of IL-4 in modulating the immune response to a conjugate oxycodone vaccine in mice [164]. The authors demonstrated that depleting IL-4 enhanced vaccine efficacy by increasing early antibody-secreting cell responses, especially oxycodone-specific IgG titers, without altering somatic hypermutation or affinity maturation. Notably, pre-immunization levels of IL-4 negatively correlated with antibody responses, suggesting IL-4 may serve as a predictive biomarker for vaccine efficacy in future OUD immunization strategies.

Other cytokine pathways have not been explored in SUD vaccine studies, but some cytokines’ immunomodulatory roles suggest potential applications. For instance, IL-21 supports Tfh and germinal center responses and can improve antibody affinity [165,166]. IL-12 or type I IFNs may be considered in transient or locally delivered formats to bias Th1/Tfh programs as systemic use has toxicity constraints [167,168,169,170]. Granulocyte-macrophage colony-stimulating factor (GM-CSF) can recruit APCs, but outcomes vary by platform and dose [171,172]. Broad IL-10 or transforming growth factor β (TGF-β) modulation is conceptually interesting but regulatory and pleiotropic, and they may be best reserved for context-limited and local rather than systemic uses (e.g., brief IL-10 blockade at immunization, targeted TGF-β to steer IgA in mucosal settings) [173,174,175,176,177].

Altogether, the classes of adjuvants discussed (aluminum salts, emulsions, TLR agonists, protein-based adjuvants, and cytokine modulators) comprise a diverse and mechanistically rich toolkit for strengthening vaccine-induced immunity against SUDs. Each platform offers unique advantages, ranging from the regulatory familiarity of Alum to the tailored immune polarization achievable with cytokine modulation. Nevertheless, the effectiveness of a given adjuvant is not universal. As emerging data reveal, identical adjuvants can produce divergent outcomes depending on the target drug, hapten structure, and immunological context. These observations underscore the importance of understanding not only how adjuvants work, but also when and for which drug targets and/or delivery system they work best. In the following sections, we explore the sources of this variability, integrating comparative study results and mechanistic insights to uncover why certain adjuvants succeed in one context yet falter in another.

## 3. Variability of Adjuvant Performance Across Drug Classes

Although the immunostimulatory properties of adjuvants are well established in immunotherapies against cancer or infectious diseases, their performance in SUD vaccines has been notably variable. In this section, we examine evidence from published studies revealing that adjuvants do not elicit consistent immune responses across different drug targets and delivery systems. Vaccines targeting different drugs and using different delivery vehicles often respond differently to the same adjuvant formulation, producing divergent outcomes in terms of antibody titers, affinity, and functional protection. These findings advocate systematic or head-to-head comparisons of adjuvants and drug-specific optimization strategies, as assumptions based on success in one context may not translate to another. Rather than searching for a default or universal solution, adjuvant selection in SUD vaccine development requires careful validation within each drug class.

Several studies have compared the effects of different adjuvants within the same vaccine platform. For instance, Zhao et al. evaluated the efficacy of different TLR agonists and their combinations in the context of a nanoparticle-based nicotine vaccine (NanoNicVac) [49]. Three TLR agonists, MPLA, R848, and CpG ODN 1826, and their combinations were efficiently encapsulated into the nanoparticle. Among the adjuvants tested, the combination of MPLA and R848 led to the highest anti-nicotine antibody titer with superior affinity and most effectively diminished nicotine entry into the brain of mice. In the following study, Hu et al. evaluated the effect of Alum as an adjuvant for NanoNicVac [178]. Surprisingly, Alum did not enhance the vaccine’s immunogenicity or its ability to reduce brain nicotine levels in mice. Instead, Alum disrupted the structural integrity of NanoNicVac, impeded its release and cellular uptake, and increased off-target anti-carrier (KLH) antibody responses. These results suggested that Alum might not be suitable for nanoparticle-based vaccine formulations. Combining the results from these two studies, the TLR agonist combination of MPLA and R848 emerged as the optimal adjuvant formulation for NanoNicVac. Alum disrupted nanoparticle structure and failed to enhance immunogenicity, rendering it unsuitable for this nanovaccine platform. Walter et al. adapted the same nanoparticle platform for an oxycodone vaccine and evaluated formulations incorporating different carrier proteins and adjuvants [80]. Vaccines formulated with MPLA and R848 underperformed compared to those adjuvanted with Alum. The vaccine candidate using KLH as the carrier protein and Alum as the adjuvant induced higher antibody titers, more effective oxycodone sequestration, and greater reduction in oxycodone’s analgesic effect than its MPLA + R848 counterpart. It is interesting to note that the MPLA + R848 combination, which demonstrated optimal efficacy in NanoNicVac, did not achieve comparable results in the oxycodone vaccine using the same nanoparticle platform. Conversely, Alum, which was ineffective and even disruptive in the nicotine vaccine, proved to be the superior adjuvant in the oxycodone formulation, eliciting stronger immune responses and better functional outcomes. These contrasting outcomes underscore the importance of context-specific adjuvant evaluation, even within a shared delivery system. These results also suggest that adjuvant efficacy can hardly be generalized across different drug targets.

In another striking example, emulsion-based adjuvants exhibited similarly divergent performance depending on the target drug and the delivery platform. Madge et al. investigated several designs and adjuvant strategies for fully synthetic, peptide-based anti-cocaine nanovaccines [102]. Their core immunogen consisted of a cocaine hapten chemically coupled to the universal T-helper epitope PADRE, which was then tethered to various self-assembling and self-adjuvanting peptides. In the initial proof-of-concept, this construct (COC-1), administered alone or emulsified in Complete Freund’s Adjuvant (CFA), elicited robust anti-cocaine IgG titers and effectively attenuated cocaine-induced hyperlocomotion in mice. To improve both delivery and intrinsic adjuvanticity, the authors next engineered two self-adjuvanting liposomal platforms: a polyleucine-based liposomal vaccine (COC-3L) and a cyclic lipopeptide variant (COC-4L). These were compared head-to-head with the hapten-PADRE immunogen without the peptide sequence (COC-5) formulated in CFA or in the clinically approved O/W emulsion MF59 (COC-5 + CFA vs. COC-5 + MF59). COC-3L and the MF59-adjuvanted formulation (COC-5 + MF59) induced the highest antibody titers and conferred the greatest behavioral protection. COC-5 + MF59 outperformed both COC-5 + CFA and the original COC-1 construct. These findings underscore MF59′s promise in synthetic peptide-based cocaine vaccines. On the other hand, Pravetoni and colleagues assessed an oxycodone conjugate vaccine, OXY(Gly)_4_-KLH, across several adjuvant formulations [39,103,179]. They found that only Freund’s adjuvant and Alum elicited robust antibody responses. Neither the combination of Alum and MPLA, MPLA alone, nor MF59 produced comparable immunogenicity. These observations are markedly different from the work by Madge et al., who reported that MF59 enhanced the efficacy of synthetic peptide-based nanovaccines against cocaine, outperforming Freund’s adjuvant. The ineffectiveness of MF59 to potentiate the oxycodone vaccine, despite its demonstrated potency in the cocaine system, highlights a critical inconsistency in adjuvant performance across different drug targets and delivery systems. These divergent outcomes underscore that adjuvant efficacy is not universally transferable between SUD vaccines. Consequently, there is a clear need for rational, drug-specific and carrier-specific adjuvant optimization, which implies designing or selecting immune stimulators tailored to the physicochemical and immunological properties of each hapten-carrier construct and delivery strategy to achieve consistent, high-level protection.

Although cross-study interpretation was performed to identify patterns in adjuvant performance, several methodological limitations should be acknowledged. Small animal models were commonly used. Sample sizes were often limited, and several studies included only one sex. Behavioral endpoints varied, and few studies conducted head-to-head adjuvant comparisons within matched vaccine backbones. To improve comparability, core immunogenicity outcomes should be prespecified, including antibody titer, affinity, isotype distribution, and, if feasible, Tfh and germinal center indices. Functional pharmacokinetic and pharmacodynamic measures should be included to link antibody responses with drug sequestration and behavior. Behavioral endpoints and assessment windows should be standardized, and sample-size justification should be provided. When feasible, adjuvants should be evaluated using the same carrier, hapten, and dosing schedule.

The next section will explore the mechanistic underpinnings of the divergent outcomes highlighted above. First, we will examine why the TLR agonist pair MPLA + R848 markedly enhanced the immunogenicity of NanoNicVac, consider how Alum may compromise nanocarrier integrity, and analyze why the same MPLA + R848 combination failed in an oxycodone formulation built on the same platform. Second, we will evaluate why MF59 outperformed Freund’s adjuvant in a peptide-based cocaine vaccine yet showed limited benefit in an oxycodone conjugate vaccine, where Alum and Freund’s adjuvant proved more effective. By exploring these case studies, our aim is not to prescribe a formulaic, substance-specific adjuvant solution but rather to distill practical principles that can guide the rational optimization of adjuvant formulations.

## 4. Mechanistic Insights Linking Drug Biology to Adjuvant Efficacy

**Figure 2:**
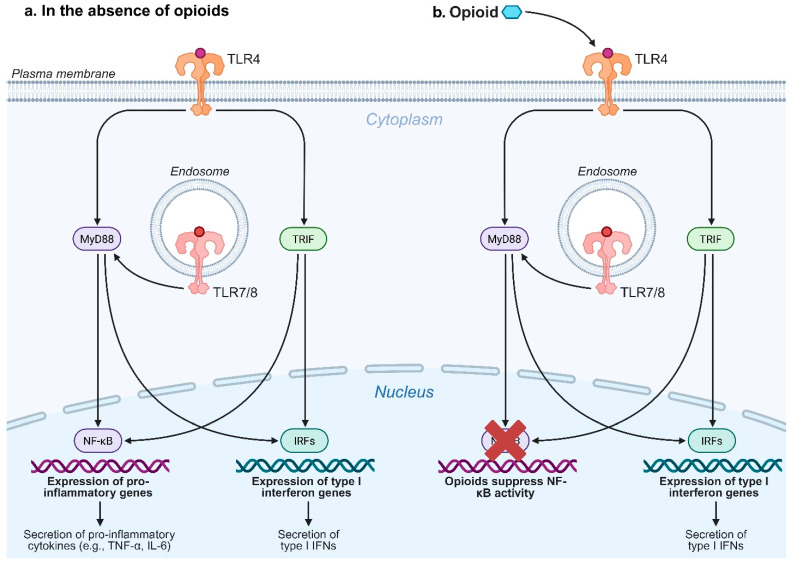
Modulation of TLR signaling by opioids. (**a**). In the absence of opioids, ligand engagement of TLR4 recruits the adaptor proteins MyD88 and TRIF, whereas TLR7/8 signals exclusively through MyD88. These signaling pathways lead to the NF-κB-dependent expression of pro-inflammatory cytokines (e.g., TNF-α, IL-6) or activate IRFs that induce type I IFN genes. (**b**). Opioids non-competitively attenuate the TLR4-induced NF-κB activation (red cross), reducing pro-inflammatory cytokine production. Arrows indicate direction of signal propagation. This figure was created in BioRender.com (modified from the template TLR Signaling Pathway).

In addition to acknowledging adjuvant variability, mechanistic insights help guide the design of new formulations and predict which immunological barriers a given substance may present. Here, we explore several expanded dimensions of drug-adjuvant and carrier-adjuvant interaction. Zhao et al. demonstrated that a nanoparticle-based nicotine vaccine formulated with MPLA + R848 achieved exceptional potency in terms of immunogenicity and pharmacokinetic outcome [49]. MPLA and R848 target TLR4 and TLR7/8, respectively, and the highest immune efficacy elicited by their combination could be mechanistically explained by their engagement of distinct signaling pathways, potentially resulting in additive or synergistic immune activation (Figure 2). Although TLR4 can recruit either the MyD88 or TRIF adaptor proteins, MPLA seems to be a TRIF-biased agonist in mice, preferentially steering the signal through the TRIF pathway [180]. By contrast, R848 binds TLR7/8 and signals predominantly via the MyD88 adaptor [181]. Engaging both the MyD88- and TRIF-dependent signaling pathways have been demonstrated to broaden the innate cytokine milieu, simultaneously driving type I IFNs and IL-12p70 alongside pro-inflammatory MyD88-dependent cytokines such as TNF-α and IL-6 [182,183,184]. This co-activation promotes dendritic cell maturation and facilitates T-helper polarization, resulting in more potent and qualitatively superior adaptive response. 

Hu et al. reported that aluminum salt compromised the structural integrity of NanoNicVac [178]. NanoNicVac consists of a PLGA core encased in a liposomal shell functionalized with nicotine hapten-KLH conjugates. Transmission electron microscopy revealed that raising the Alum-to-NanoNicVac mass ratio progressively eroded this lipid envelope. At a 1:1 ratio, the lipid layer was nearly abolished. Although the authors did not specify which aluminum salt was used, the physicochemical profile most consistent with such damage is that of aluminum oxyhydroxide (AlOOH), or Alhydrogel®. Its strongly positive ζ-potential at physiological pH can electrostatically desorb or rearrange zwitterionic phospholipid headgroups, undermining lipid shell integrity [185,186]. Moreover, the rigid, high-aspect-ratio nanofibers of AlOOH can perforate the lipid film, an effect corroborated by monolayer studies documenting decreased compressibility on contact. Collectively, these charge- and shape-mediated interactions offer a mechanistic rationale for the Alum-induced delamination of the liposomal corona observed in NanoNicVac. Minimizing Alum’s residence time in the nanoparticle formulation could mitigate such damage. Walter et al. mixed Alum with the oxycodone nanovaccine immediately before administration in mice and reported no discernible disruption of nanoparticle architecture [80].

The MPLA + R848 adjuvant combination that elicited the highest immunogenicity in NanoNicVac did not replicate its effect in the oxycodone vaccine evaluated by Walter et al. [80]. One plausible explanation is the crosstalk between opioids and TLR signaling pathways [187]. As depicted in Figure 2, MPLA is derived from lipopolysaccharide (LPS) and acts primarily through TLR4 activation [188]. However, it has been reported that several opioids interact with TLR4 and attenuate LPS-induced responses. In a study by Stevens et al. [189], although morphine and fentanyl triggered only slight TLR4 activation on their own, they substantially and non-competitively suppressed LPS-induced NF-κB activation. Madera-Salcedo et al. further demonstrated that morphine inhibited LPS-stimulated TNF release from bone marrow-derived mast cells by promoting a β-arrestin-2/TNF receptor-associated factor 6 (TRAF6) complex that dampens NF-κB signaling [190,191]. Similarly, Bencsics et al. illustrated that systemic morphine dose-dependently inhibited LPS-induced TNF-α production in mice [192]. These studies suggest that oxycodone and other opioids antagonize LPS-mediated TLR4 activation. Accordingly, oxycodone vaccine formulated with MPLA yielded lower anti-oxycodone antibody titers. These findings advocate against using MPLA and other TLR4 agonists as adjuvants in vaccines targeting OUDs.

**Figure 3:**
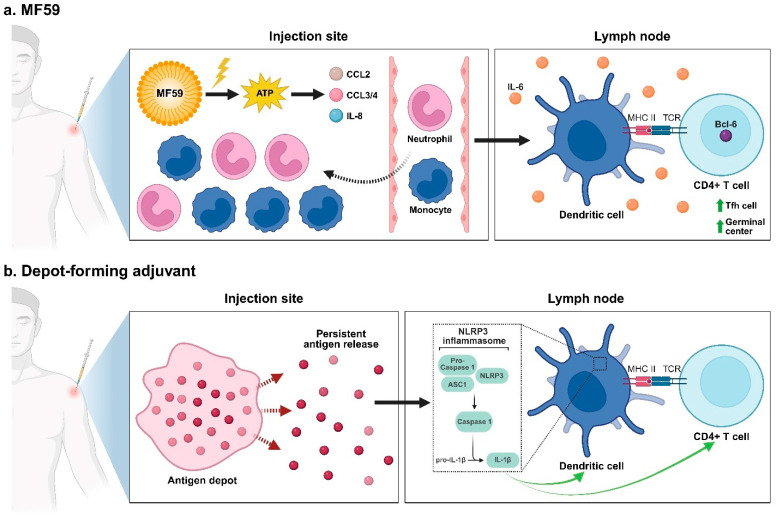
Divergent immunological pathways engaged by MF59 and depot-forming adjuvants. (**a**). MF59, a squalene O/W emulsion, provokes a transient release of extracellular ATP that elicits a burst of chemokines (CCL2, CCL3/4, IL-8). The resulting gradient rapidly attracts neutrophils and inflammatory monocytes to the injection site. Recruited monocytes differentiate into CD11c^+^ dendritic cells that migrate to the draining lymph node, where they present peptide-MHC class II complexes in an IL-6-rich milieu. IL-6 up-regulates Bcl-6 in newly primed CD4^+^ T cells, expanding the Tfh pool and amplifying germinal center B-cell reactions. (**b**). Depot-forming adjuvants retain antigen at the injection site and activate the NLRP3 inflammasome in APCs. Activated caspase-1 cleaves pro-IL-1β into mature IL-1β, which feeds back on APCs and CD4^+^ T cells to enhance immune priming and differentiation. This figure was created in BioRender.com.

Building on the comparative efficacy data presented above, we now explore the mechanistic traits that underline MF59’s context-dependent performance. As illustrated in Figure 3, MF59 is a squalene O/W nano-emulsion that does not create a classical depot. Instead, it provokes a transient release of extracellular ATP, which in turn triggers a chemokine burst of the C-C motif chemokine ligand 2 (CCL2), CCL3/4 and IL-8 without requiring NLRP3 activation [93,193,194,195,196]. The chemokine gradient leads to the rapid infiltration of neutrophils and CCR2^+^ inflammatory monocytes into the injection site. Many of these monocytes differentiate into CD11c^+^ dendritic cells and reach the draining lymph node, where they present peptide-MHC class II in an IL-6-rich milieu that favors B-cell lymphoma 6 (Bcl-6) expression in newly primed CD4^+^ T cells [95,197]. Bcl-6 is a master transcription factor that regulates Tfh cell differentiation and proliferation [198]. Tfh cells amplify germinal center B-cell reactions and are extremely important for small-peptide antigens whose intrinsic T cell help is weak. In the study by Madge et al., several peptide-based anti-cocaine nanovaccines were synthesized and evaluated [102]. While the peptides contained the universal T-helper epitope PADRE to compensate for limited T-helper engagement, this measure alone might not fully restore helper activity. Incorporating MF59 further amplified T-helper responses and markedly enhanced both the immunogenicity and protective efficacy of the peptide-based anti-cocaine nanovaccines.

In comparison, the oxycodone conjugate vaccine OXY(Gly)_4_-KLH already incorporates a highly immunogenic carrier rich in CD4^+^ epitopes, so the limiting step here shifts from T-helper activation to antigen persistence. Alum creates a long-lasting depot effect, protects the conjugate from rapid clearance, and unlike MF59, activates the NLRP3 inflammasome-mediated pathways [69,76,199]. CFA supplies a similar slow-release depot and activates the NLRP3-apoptosis-associated speck-like protein containing a caspase-recruitment domain (ASC)-caspase-1 inflammasome, which cleaves pro-IL-1β into its mature, bioactive form [200,201]. Mature IL-1β feeds back through IL-1 receptor-MyD88 signaling on both dendritic cells and responding CD4^+^ T cells (Figure 3). As aforementioned, the oxycodone conjugate vaccine does not lack T-helper involvement, while improving antigen persistence could potentially further its efficacy. The longer-lived depot effect generated by Alum and CFA precisely addressed the need for prolonged antigen presentation, resulting in higher oxycodone-specific antibody titers and stronger pharmacological blockade than MF59 in rodent models [39,103,179]. Another complicating factor for opioid vaccines is that many opioids actively modulate immune function and influence the production of various cytokines [139,202,203,204]. The immunomodulatory effects can blunt the chemokine environment critical for MF59’s adjuvanticity, contributing to its reduced efficacy in opioid vaccines.

Taken together, these mechanistic insights suggest that an adjuvant’s immunomodulatory action should bypass immune pathways suppressed by the target drug and compensate for the antigen’s remaining immunogenic deficits. Co-encapsulation of MPLA and R848 in a nicotine nanovaccine significantly improves immunogenicity and pharmacokinetic efficacy. However, the same adjuvant strategy fails for opioid vaccines as opioids blunt TLR4 signaling, rendering MPLA ineffective. In the other example, MF59 is advantageous when rapid leukocyte recruitment and Tfh expansion are needed, as in vaccines based on short peptides or self-assembling lipopeptides. Nonetheless, it offers little added value when a hapten carrier already supplies abundant T cell help. For opioid vaccines formulated by conjugating haptens to a strongly immunogenic protein carrier, adjuvants that provide a physical depot, such as Alum and Freund’s adjuvant, currently appear better suited.

Beyond drug pharmacology and adjuvant mechanisms, variability across platforms often arises from how the delivery vehicle engages innate immune sensing. In nanoparticle-based systems, particle size and interfacial chemistry influence key processes such as lymphatic trafficking, depot formation, and the presentation of danger signals [205,206,207,208]. These properties, in turn, affect how effectively the immune system is activated. When a carrier already provides innate cues or creates a depot effect, using adjuvants with complementary mechanisms is more likely to enhance immune responses than using ones with overlapping functions. This synergy is especially evident in co-delivery strategies that bring both antigen and innate agonists to the draining lymph node, where they can promote robust Tfh and germinal center responses [209,210,211]. The effectiveness of such strategies can be further improved by controlling the stoichiometry and release kinetics of the components. Compared to simple admixtures, this level of control leads to more consistent and potent outcomes. For this reason, co-encapsulation is preferred when feasible, as it ensures coordinated spatial and temporal delivery of antigen and adjuvant signals.

**Figure 4:**
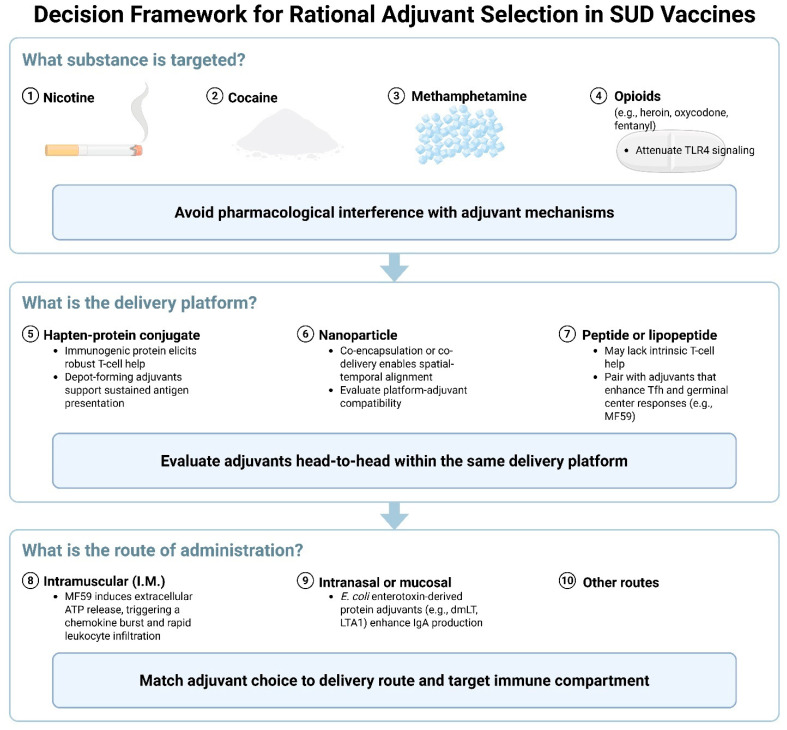
Decision framework for rational adjuvant design in SUD vaccines. The schematic outlines key decision points in the design of SUD-targeted vaccines, organized by the addictive substance targeted, the delivery platform, and the route of administration. Each design layer influences the immune context and adjuvant requirements. For instance, hapten–protein conjugates usually have strong T cell help and may benefit from depot-forming adjuvants that promote antigen persistence. Nanoparticle platforms allow co-delivery of antigen and adjuvants but require careful evaluation of carrier-adjuvant compatibility. Self-assembling peptide vaccines often require additional adjuvant support (e.g., MF59) to enhance Tfh and germinal center responses. Intramuscular delivery with MF59 can elicit chemokine bursts via ATP release, while mucosal strategies with dmLT or LTA1 support IgA induction. This figure was created in BioRender.com.

Evidently, adjuvant performance in SUD vaccines is best interpreted as conditional on both drug biology and formulation context. Findings with strong support include MF59 enhancing leukocyte recruitment and Tfh support for peptide or lipopeptide constructs and CpG in combination with Alum improving antibody response in several stimulant and nicotine models. Effects that vary by context include reduced utility of TLR4 agonists in opioid settings and limited added value from MF59 when carrier proteins already provide strong T cell help and antigen persistence is the limiting step. Positive synergies are most likely when antigen and innate cues are co-localized and mechanistically complementary rather than duplicative. Because most evidence is preclinical and head-to-head evaluations within identical backbones remain uncommon, these conclusions should be treated as evidence-weighted patterns rather than universal rules. To support context-specific adjuvant selection in SUD vaccine development, Figure 4 presents a decision framework. It integrates key design questions and considerations related to substance class, delivery platform, and route of administration.

## 5. Translational Barriers for SUD Vaccines

Human trials with nicotine and cocaine vaccines showed that immunization was able to elicit measurable anti-drug antibodies, yet clinical benefit was inconsistent and largely confined to participants who achieved higher and more durable titers. Phase 3 nicotine programs with 3′-AmNic-rEPA (NicVAX®, NCT00836199) and the pivotal multicenter cocaine study (TA-CD, NCT00969878) reported immunogenicity but did not meet primary efficacy endpoints in intent-to-treat analyses [52,60,84,212]. Signals of reduced drug use or higher abstinence rates appeared mainly in higher-titer subgroups. 

Trial design choices compound this challenge. Endpoints have varied widely, from biochemically verified continuous abstinence in nicotine studies to thrice-weekly urine benzoylecgonine testing in cocaine studies [60,84], which complicates cross-trial comparison. Future studies should prespecify responder definitions that link mechanistic thresholds to outcomes (e.g., IgG concentration and affinity targets tied to plasma drug sequestration and validated behavioral measures) and conduct power analyses accordingly. Because antibody generation requires weeks and peak titers may wane, vaccination schedules and booster timing must be aligned with high-risk windows for relapse to maintain coverage. 

Behavioral context is equally important. Partial blockade can prompt some individuals to escalate drug use to overcome antibody binding, which erodes apparent efficacy and may increase risk [38,213]. Adherence, counseling, and integration with evidence-based behavioral therapies should therefore be embedded within vaccine trials, and combination with standard-of-care pharmacotherapies considered wherever appropriate. 

Ethical and social considerations shape both trial conduct and real-world adoption. Voluntary participation should be explicit, with use in justice or correctional settings limited to opt-in models that avoid coercion or perceived compulsion [214,215,216]. Informed consent should make clear that vaccines do not guarantee abstinence and that partial pharmacologic blockade may still permit intoxication, with counseling on harm reduction and overdose risk mitigation when relevant [217,218,219,220]. Patient acceptability is influenced by clarity about expected benefits and limitations, the burden of dosing and boosters, the availability of supportive behavioral therapies, and, when appropriate, pharmacotherapies. Privacy protections for biological monitoring and drug testing data should be specified, along with plans for non-punitive management of relapses. Equity of access, cost considerations, and strategies for reaching populations with limited healthcare engagement should be addressed prospectively, including post-trial access when feasible. Community and patient input into study materials and recruitment can further improve acceptability and trust.

Practical considerations influence feasibility. Adjuvant selection affects both immunogenicity and tolerability, and certain formulations introduce additional chemistry, manufacturing, and control requirements. Programs should standardize reactogenicity monitoring, plan for lot comparability, and assess cost-effectiveness for prevention and relapse-prevention use cases, with ethical implementation emphasizing voluntary participation, transparent consent, realistic expectation setting, and attention to access and equity [38,221,222]. Taken together, lessons from nicotine and cocaine trials suggest that success will depend on predictable induction of high-quality antibody responses in a meaningful fraction of patients, rigorous linkage of these responses to clinically meaningful endpoints, and trial designs that integrate behavioral and implementation scaffolding alongside optimized vaccine constructs.

## 6. Conclusions and Future Directions

Adjuvant incorporation is often the determinant that converts a modest anti-drug immunogen into a viable vaccine candidate. More than twenty years of preclinical and clinical studies on vaccines against SUDs reveal several recurring principles. First, no adjuvant exhibits universal efficacy. For instance, one formulation that performs well against nicotine or cocaine may prove inadequate against opioids. Second, drug chemistry and pharmacology directly influence immune outcomes. For example, opioids that attenuate TLR4 signaling can diminish the potency of TLR4-targeted agonists such as MPLA. Third, structural compatibility is essential. Alum can destabilize lipid-shelled nanocarriers. Finally, yet importantly, combination or self-adjuvating platforms consistently outperform single agents when they co-activate complementary pathways or co-localize antigen and danger signals within the same molecular scaffold. Co-encapsulation of MPLA and R848 within the same nanoparticle platform enables NanoNicVac to achieve greater efficacy than formulations with a single adjuvant. These principles are supported chiefly by preclinical evidence. Within the smaller human literature, efficacy signals have been confined to higher-titer subgroups and overall results have been mixed. 

In addition to these design principles, adjuvant selection should match the delivery route and the intended immune compartment (Figure 4). When exposure occurs at mucosal surfaces, such as inhaled nicotine, intranasal or oral vaccination can be considered. Adjuvants such as dmLT or LTA1, when administered by mucosal routes, have induced local secretory IgA that complements systemic IgG. For parenterally used drugs, such as injected opioids, the emphasis remains on eliciting durable, high-affinity IgG in the circulation. 

Precision adjuvant selection can be put into practice through parallel, standardized head-to-head screenings conducted within matched vaccine backbones for each target drug class. A common data frame should include antibody titer, affinity, isotype distribution, and, if feasible, Tfh and germinal center indices. Pharmacokinetic and pharmacodynamic readouts that link antibody profiles to drug sequestration and behavior should be captured. These data can then be combined with predictive biomarkers to guide regimen choice and boosting. For example, baseline IL-4, innate transcriptional signatures, and early titer or affinity data can identify likely responders. Prespecified immunogenicity thresholds can trigger adaptive booster timing and composition. Because many preclinical and available clinical trials did not report sex-stratified outcomes or were not powered to detect them, sex should be considered a limitation of the current evidence base and a prespecified variable for future studies.

Attention to platform and adjuvant compatibility is equally important. Design-of-experiments evaluation of adsorption, release, stability, and depot behavior with the intended carrier should be routine. Pairings known to be incompatible, such as alum with fragile lipid-shelled particles, should be avoided. Clinical translation is strengthened when composite endpoints are prespecified that pair clinical outcomes with immunologic and pharmacologic targets (e.g., anti-drug IgG concentration and affinity targets, an isotype pattern linked to efficacy, and a predefined reduction in plasma drug exposure after a standardized challenge). Manufacturing scale-up, lot comparability, and reactogenicity monitoring appropriate to each adjuvant class should be planned prospectively. Enrollment and analysis plans that allow sex-stratified reporting when adequately powered improve interpretability. Implementation is expected to be most effective when vaccination is accompanied by behavioral therapies and, when appropriate, pharmacotherapies, with voluntary use, transparent consent, realistic expectations, and attention to access and equity.

## Figures and Tables

**Table 1:** Summary of aluminum salt-based adjuvants used in SUD vaccines.

Adjuvant	Vaccine Target	Species Tested	Performance	References
Aluminum hydroxide (AH)	Nicotine (NicVAX®, Niccine®), Cocaine (TA-CD)	Human	Safe with moderate immunogenicity; limited efficacy in sustaining abstinence; high responders showed partial benefits	Hatsukami et al. [83], Hoogsteder et al. [58,60], Tonstad et al. [59], Martell et al. [52], Kosten et al. [84]
	Oxycodone	Mouse	Alum elicited a more robust antibody response than the TLR agonists R848 and MPLA	Walter et al. [80]
	Methamphetamine (IC_KLH_-SMO9), morphine (KLH-6-SM), nicotine (trivalent)	Rat	Effective in terms of immunogenicity and protective efficacy; used in multivalent constructs	Rüedi-Bettschen et al. [77], Kosten et al. [78], de Villiers et al. [81]
	Multivalent against fentanyl, carfentanil, oxycodone, heroin, methamphetamine, and their analogs or metabolites	Mouse, rat	Effective in inducing independent antibody responses against the respective targets	Song et al. [82]
Alum adjuvant not specified	Oxycodone (6OXY(Gly)_4_-KLH)	Mouse, rat	Effective against both oxycodone and hydrocodone	Pravetoni et al. [79]

**Table 2:** Summary of emulsion-based adjuvants evaluated for SUD vaccines.

Adjuvant	Vaccine Target	Species Tested	Performance	References
Freund’s Adjuvant	Fentanyl (FEN-BGG), Cocaine (COC-BSA), Nicotine (3′-AmNic-rEPA)	Mouse, rat	Strong antibody responses; not approved for human use due to safety	Torten et al. [96], Fox et al. [97], Pravetoni et al. [98]
Sigma Adjuvant System® (SAS)	Methamphetamine (MH6), Nicotine (triAM1(Gly)2)	Mouse	Elicited high titers and strong affinity; trivalent formulation outperformed monovalent	Moreno et al. [99], Miller et al. [100], Collins and Janda [101]
MF59	Cocaine (COC-5+MF59), Oxycodone (OXY(Gly)4-KLH)	Mouse	Enhanced efficacy in peptide-based vaccines but not in conjugates	Madge et al. [102], Robinson et al. [103]
AS03	Nicotine (AM1-TT)	Mouse, rat	Enhanced immunogenicity in mice and rats; reduced nicotine self-administration in rats	Moreno et al. [104]

**Table 3:** Summary of TLR agonists employed as adjuvants in SUD vaccines.

Adjuvant	Vaccine Target	Species Tested	Performance	References
TLR2 agonist (Pam3CAG)	Nicotine	Mouse	Effective when co-administered with MPLA	Lockner et al. [120]
TLR3 agonist (dsRNA)	Heroin	Mouse	Effective when co-formulated with AH	Hwang et al. [122]
TLR4 agonist (MPLA)	Nicotine, Heroin, Oxycodone, heroin and fentanyl bivalent	Mouse, rat	Synergistic with R848 for nicotine; ineffective for oxycodone due to TLR4 suppression; effective in bivalent vaccine when co-formulated with AH	Zhao et al. [49], Matyas et al. [113], Walter et al. [80], Barrientos et al. [119]
TLR 4 agonist (GLA-SE)	Methamphetamine	Mouse	Higher efficacy compared to AH	Stevens et al. [123]
TLR5 agonist (entolimod)	Methamphetamine	Mouse, rat	Effective when co-administered with AH	Haile et al. [118]
TLR7/8 agonist (R848)	Nicotine, Oxycodone	Mouse	Strong synergy with MPLA in nicotine vaccines	Zhao et al. [49], Walter et al. [80]
TLR7/8 agonist (UM-3006)	Fentanyl	Mouse	Effective when co-conjugating hapten and UM-3006 to carrier; synergizes with AH to increase efficacy	Powers et al. [121]
TLR9 agonist (CpG ODN)	Fentanyl, Heroin, Cocaine, Methamphetamine	Mouse, rhesus monkey	Synergizes with AH to increase efficacy	Bremer et al. [114,115], Hwang et al. [122], Kimishima et al. [116], Hossain et al. [117]

**Table 4:** Summary of protein-based adjuvants in the development of vaccines for SUDs.

Adjuvant	Vaccine Target	Species Tested	Performance	References
Flagellin	Cocaine (GNE-FliC)	Mouse	Dual role as carrier and adjuvant; synergizes with AH to increase efficacy	Lockner et al. [136]
*E. coli* enterotoxin-derived dmLT and LTA1	Fentanyl	Mouse	Efficacious; mucosal routes induced IgA	Stone et al. [63]
B subunit of cholera toxin (CTB)	Cocaine (TA-CD)	Human	Safe with moderate immunogenicity; limited efficacy in sustaining abstinence; high responders showed partial benefits	Kosten et al. [84]
Peptide containing a B cell epitope (YKQGGFLGL) and a conformationally biased C5a receptor agonist (YSFKPMPLaR)	Nicotine	Rat	Dual role as carrier and adjuvant; effective without external adjuvants	Sanderson et al. [137]
Self-assembling peptide nanofiber (KFE8)	Cocaine	Mouse	Dual role as carrier and adjuvant; effective without external adjuvants	Rudra et al. [138]

**Table 5:** Summary of cytokine modulators and IL-4 neutralization strategies in SUD vaccine formulations.

Adjuvant	Vaccine Target	Species Tested	Performance	References
IFN-γ	Oxycodone	Mouse	Enhanced titers and brain protection when paired with TLR agonists	Bian et al. (manuscript in preparation)
IL-4 neutralization (anti-IL-4 mAb)	Oxycodone, Fentanyl	Mouse	Enhanced IgG2a and IgG3, improved germinal center response and protection	Laudenbach et al. [162], Crouse et al. [163,164]

## Data Availability

No new data were created or analyzed in this study. Data sharing is not applicable to this article.

## Abbreviations

AH	aluminum hydroxide (aluminum oxyhydroxide, AlOOH) adjuvant
AlOOH	aluminum oxyhydroxide
Alum	aluminum salt-based adjuvants in general or when the specific salt is unknown
AP	aluminum phosphate adjuvant
APC	antigen-presenting cell
ASC	apoptosis-associated speck-like protein containing a CARD
AS03	squalene oil-in-water emulsion adjuvant
ATP	adenosine triphosphate
Bcl-6	B-cell lymphoma 6 (Tfh lineage-defining transcription factor)
BGG	bovine gamma globulin
BSA	bovine serum albumin
CARD	caspase-recruitment domain
CCL2	C-C motif chemokine ligand 2
CCL3/4	C-C motif chemokine ligand 3/4
CCR2	C-C motif chemokine receptor 2
CFA	Complete Freund’s Adjuvant
COC	cocaine
CpG ODN	CpG oligodeoxynucleotide (TLR9 agonist)
CRM_197_	cross-reactive material 197 (nontoxic diphtheria toxin mutant)
DAMP	damage-associated molecular pattern
DC	dendritic cell
dmLT	double-mutant *E. coli* heat-labile enterotoxin (LT)
dsRNA	double-stranded RNA (TLR3 agonist)
FEN	fentanyl
GLA-SE	glucopyranosyl lipid A in squalene emulsion (TLR4 agonist)
IFN	interferon (e.g., IFN-β, IFN-γ)
IgG	immunoglobulin G (e.g., IgG2a, IgG3)
IL	interleukin (e.g., IL-1β, IL-6, IL-12p70)
I.V.	intravenous
KLH	keyhole limpet hemocyanin
LP	liposome
LPS	lipopolysaccharide
LT	*E. coli* heat-labile enterotoxin
LTA1	A1 domain of *E. coli* heat-labile enterotoxin (LT)
MF59	squalene oil-in-water emulsion adjuvant
MHC	major histocompatibility complex
MPLA	monophosphoryl lipid A (TLR4 agonist)
MyD88	myeloid differentiation primary response protein 88 (TLR adaptor)
NanoNicVac	nicotine nanovaccine with a PLGA core and liposomal shell
NF-κB	nuclear factor kappa-light-chain-enhancer of activated B cells
NLRP3	NOD-like receptor protein 3 inflammasome
NP	nanoparticle
O/W	oil-in-water (emulsion)
OUD	opioid use disorder
OXY	oxycodone
PADRE	Pan DR-binding epitope (universal CD4^+^ T-helper epitope)
PAMP	pathogen-associated molecular pattern
PLGA	poly(lactic-co-glycolic acid)
PRR	pattern-recognition receptor
R848	resiquimod (TLR7/8 agonist)
rEPA	recombinant Pseudomonas exoprotein A
SAS	Sigma Adjuvant System (O/W emulsion)
SUD	substance use disorder
Th1/Th2	T helper type 1/type 2
Tfh	T follicular helper (cell)
TLR	toll-like receptor
TNF-α	tumor necrosis factor-alpha
TRAF6	TNF receptor-associated factor 6
TRIF	TIR-domain-containing adaptor inducing IFN-β (TLR adaptor)
TT	tetanus toxoid
UM-3006	small-molecule TLR7/8 agonist
W/O	water-in-oil (emulsion)
